# Stigmatization of patients with mental disorders: a comparative study of nurses in forensic psychiatry and inpatient settings

**DOI:** 10.3389/fpsyt.2024.1440917

**Published:** 2024-08-15

**Authors:** Barış Kılıç-Demir, Selma Çilem Kızılpınar

**Affiliations:** Department of Psychiatry, Ministry of Health Adana City Training & Research Hospital, Adana, Türkiye

**Keywords:** forensic psychiatry, nurses, mental disorders, community psychiatry, criminals, stigmatization

## Abstract

**Background:**

Forensic psychiatric patients require specialized care due to the unique challenges in forensic settings. Negative attitudes and beliefs towards mentally disordered offenders can lead to discrimination against patients and are related to worse outcomes. Forensic psychiatric nurses play a crucial role in the treatment of these patients.

**Aim:**

This study aimed to investigate the perceptions, attitudes, and beliefs of forensic psychiatric nurses and general medicine nurses towards psychiatric patients and to compare them between nurse groups.

**Method:**

The study was conducted with 46 nurses working in the High-Security Forensic Psychiatry Clinic (Mean age: 35.46, SD= 7.16) and 58 nurses working in other inpatient settings (Mean age:36.28, SD=8.78) aged between 18-65 between December 2020 and April 2021 in Turkey. Nurses in the forensic psychiatry clinic were required to have at least 6 months of experience in the clinic.

**Results:**

Forensic psychiatric nurses exhibited more positive attitudes towards patients (p<.0001), showing lower tendency to be socially distant (p=.009), higher trust (p<.0001), higher willingness to treat (p<.0001), lower tendency to perceive patients as threatening (p=.004), and more general positive attitudes. Significant relationships were found between some of the stigma-related scales we used and certain factors. For forensic nurses, being male (p=0.043) and single (p=,025), working long hours (p=.047), and having fewer children (p=.005) were related to more negative perceptions about delinquents. Insufficient knowledge about forensic psychiatry was linked to negative beliefs about mental disorders (p=0.017) and specifically the curability of mental disorders (p=0.008). Having more siblings was related to higher embarrassment about mental disorders (p=.043). For general nurses, having first-degree relatives who receive psychiatric treatment was related to perceiving patients as threatening (p=.021)) and negative perceptions about delinquents (p=.007). Being older was related to more positive beliefs about mental patients’ dangerousness (p=.026). Having more siblings was associated with higher trust toward patients (p=0.002).

**Conclusions:**

These findings emphasize the importance of addressing stigmatization among healthcare professionals, particularly forensic psychiatry nurses and general nurses. Providing comprehensive training about mental disorders and forensic psychiatry and promoting empathy and understanding can enhance the quality of care for patients with mental illnesses and contribute to better mental health outcomes for society.

## 1 Introduction

Patients with mental disorders often experience stigmatization, social distancing, exclusion, and rejection from the general public (1, 2). Some people have distorted beliefs that a mental disorder is a sign of character weakness, deviance, low intelligence, unreliability, and incompetence and often associate these conditions with violence and unpredictability (3). Consequently, such negative attitudes, beliefs, and perceptions contribute to impaired social skills and loss of self-esteem (4–6), reduced seeking behavior for treatment among patients, and ultimately lead to negative outcomes in mentally disordered patients (7). Conversely, it has been shown that positive attitudes towards patients with psychiatric disorders, such as being accepting, supportive, and tolerant, may allow the patient to feel relaxed, integrate with society, and ensure higher participation in treatment and care (8).

Studies revealed stigmatization of psychiatric patients not only from the general public but also from health professionals and mental health professionals (2, 6, 9–11). Therefore, it is necessary to consider the attitudes of health professionals, especially mental health professionals, in this regard. Studies commonly conducted on mental health professionals indicate more positive beliefs and attitudes than the general public (12, 13), but some publications present contradictory findings (10, 14). Despite the extensive literature on public stigma towards psychiatric patients, research specifically involving mental health professionals remains limited (14, 15). Given that the attitudes, beliefs, and perceptions of mental health professionals significantly impact patient care, investigating stigmatization among this group is essential.

Treatment of forensic psychiatry patients is important in terms of society’s mental health. Effective care of forensic psychiatry patients reduces harm to the environment and improves societal mental health (16, 17). The approach to forensic psychiatric patients has differences from the general medical services and even mental health settings. Moreover, forensic settings have significant environmental, relational, and clinical challenges (18–22). Treatment and protection in forensic psychiatric settings require multidisciplinary teamwork and forensic psychiatric nursing is an important part of treatment. Nurses working in forensic psychiatry settings are known as forensic psychiatric nurses (FPNs) (23). FPNs must be knowledgeable about the physical, psychological, social, economic, and cultural status of the forensic psychiatric patient from an integrative perspective. They must be capable of supporting and encouraging patients to develop social and practical life skills (18, 24) and managing problematic and challenging patient behaviors (25). In this regard, we believe that researching stigmatization among FPNs can significantly enhance forensic psychiatric care. To achieve this, our plan involves identifying potential factors linked to stigma among FPNs and constructing a conceptual framework by comparing them with non-FPNs.

The distinctive properties of the forensic psychiatric nursing profession bring vulnerabilities in treatment. For example, negative attitudes towards patients may influence the treatment more in forensic psychiatry settings compared with general medical settings. Stigmatization of and negative attitudes and negative stereotypical beliefs towards mentally disordered patients lead to discrimination against the patients. Although there are some studies on psychiatric nursing in general (26, 27), there is limited research on the attitudes of forensic psychiatric nurses (28). Studies on stigma in the nursing profession indicated that forensic nurses might question their health professional identities with their negative attitudes (29) and negative attitudes could affect their work engagement (30). There was no study comparing general nurses and FPNs in terms of attitudes and beliefs toward mentally disordered offenders and perception toward delinquents (10). FPNs’ negative attitudes, beliefs, and perceptions may lead to poor care and diminish the provision of competent and compassionate care to patients. For these reasons, it is essential to conduct studies on FPNs’ attitudes and to understand the factors that may influence these attitudes.

Stigmatization and culture are indistinguishable from each other. It is plausible that the stigmatization among health professionals could be influenced by their culture and social structure (31, 32). Cultural beliefs, values, and norms are important regarding the stigmatization (33). While there are various studies on medical professionals in the Middle East (34), Europe (12), and Asia (32), we believe it is essential to conduct research specific to our country to understand the unique characteristics of Turkish society regarding stigmatization. Turkish society exhibits both cultural similarities and differences with neighboring regions due to factors such as geographical proximity and shared religious values. Turkey is undergoing a significant cultural transformation from traditional rural to modern urban lifestyles, leading to a wide spectrum of attitudes and values within the society (35). Although there are studies investigating public attitudes towards mentally disordered patients in Turkish society (35), studies on professionals are limited. Therefore, there is an urgent need for more data on stigmatization among health professionals in Turkey. Our study is the first study investigating stigmatization in forensic psychiatric nurses in Turkey.

The present study is crucial as it compares forensic psychiatry nurses with general medical nurses. The study aimed to detail findings about beliefs and attitudes towards psychiatric disorders and patients and perceptions of delinquents and to compare the levels of stigmatization between FPNs and general setting nurses (non-FPN).

## 2 Materials and methods

### 2.1 Settings and subjects

The study sample consists of nurses who were employed in high-security forensic psychiatric units (FPN, n=46) and nurses who were engaged in other inpatient units (non-FPN, n=58) in the same hospital between December 2020 and April 2021 in Adana, Turkiye. We included the nurses aged 18 to 65 who worked either in forensic psychiatry services or general inpatient services and volunteered to participate in our study. In total, 46 FPNs were invited, and all participated and completed the scales. We recruited all nurses in the forensic psychiatry hospital during the study period. Additionally, we invited 65 non-FPNs. However, eight of 65 were excluded from the study because they did not complete most of the scales.

The FPN group included in our study did not receive any specialized training in forensic psychiatry nursing nor participate in any lectures or scientific activities in academic settings. However, they had experience working in a high-security forensic psychiatric unit for different durations (for at least 6 months). FPNs provide treatment for offenders who do not have criminal responsibility due to mental disorders, follow up with patients during their shifts in high-security forensic psychiatry units, and are involved in all phases of treatment in our hospital. FPNs typically care for patients diagnosed with psychotic disorders, bipolar disorder, and intellectual disabilities. The severity of the symptoms of the patients differs from mild to severe. The non-FPN group included nurses who have not worked in any psychiatric settings before and who had been recently employed in general inpatient units. While constructing the population for the FPN group, all nurses with at least 6 months of experience in forensic psychiatry services were invited to participate. For the non-FPN group, we individually visited all non-psychiatric adult patient settings, and nurses who were currently employed in these wards were invited to participate. Participation was based on volunteerism and no participant refused to participate in the study. We arranged a meeting with volunteers who met the inclusion criteria in the hospital’s social areas. The researchers first administered a sociodemographic data form, followed by a brief explanation of the other scales. Participants were then asked to complete the scales by marking their responses. All scales were completed during a single 30-minute session. FPNs and non-FPNs were compared in terms of beliefs and attitudes about psychiatric disorders and mentally disordered patients, perceptions towards delinquents, and associated factors. Perceptions, attitudes, and beliefs about mental disorders and patients all reflect stigmatization; therefore, throughout the text, the term “stigmatization” will occasionally be used in place of these terms. These expressions will be used instead of “stigmatization” only when a particular emphasis is needed. A sociodemographic data form, the Beliefs Toward Mental Illness Scale (BTMI), the Nurse’s Attitude Towards Forensic Psychiatric Patients Scale (NAFPPS), and the Perceptions Toward Criminals Scale (PTCS) were administered to all participants. Ethics committee approval was given by the hospital Ethics Committee (with the date 18.11.2020 and decision number 1139). The procedures used in the current study are designed according to the Declaration of Helsinki. Informed consent was obtained from all participants.

### 2.2 Assessment instruments

The nurses’ sociodemographic characteristics and occupational characteristics in the forensic psychiatry service were evaluated by the sociodemographic data form. The questions about their views on forensic psychiatry (their knowledge of the legal regulations in the Turkish Legal System for forensic psychiatric patients) and their position on working in forensic psychiatric settings were added to the socio-demographic data form. For this purpose, “Do you think you have enough knowledge about the legal aspect of forensic psychiatry?” was asked of all participants. “Would you like to work in the forensic psychiatric settings?” was asked of the non-FPN group.

The Perceptions Toward Criminals Scale was developed to measure the perceptions of professionals about criminals in the Turkish language by Gonultas et al. (36). It is a 5-point Likert-type scale that has two factors (perceptions towards criminals’ personality traits/moral characteristics and perceptions towards criminals’ social networks) and 12 items. The total score varies between 12-60. The scale contains negative statements about criminals. High scores indicate high negative perceptions. In this scale, the first factor explained 32.42% of the total variance, while the second factor explained 20% of the total variance (52.42% in total). The Cronbach’s alpha value of the first factor was 0.85, the second factor was 0.73. The validity analysis showed a good fit (*X* ^2^/SD=2.43; RMSEA=0.68; GFI=0.935; AGFI=0.905; NFI=0.902; CFI=0.94). The scale is the offenders as ‘criminals’. Thus, we used the original name of the scale when we mentioned the scale characteristics. However, this expression stigmatized the participants, so we referred to them as ‘delinquents’ instead of ‘criminals’ in the text.

The Beliefs Toward Mental Illness Scale was developed to measure professionals’ beliefs about mental disorders, and validity and reliability studies were conducted in many languages (37). The Turkish adaptation study was conducted by Bilge and Cam (38). It is a 6-point Likert-type scale (scored between 0-5), that has 3 factors: Dangerousness (8 items), Embarrassment (2 items), and Social Dysfunction and Incurability (11 items). The total score varies between 0-105. The statements in the scale include negative beliefs about mental disorders. The dangerousness subscale indicates beliefs about mental disorders and patients being dangerous. The social dysfunction and incurability subscale contains beliefs that mental disorders negatively affect interpersonal relationships and that it is incurable as a result. The embarrassment subscale states mental disorders are something to be ashamed of. High scores on the scale indicate high negative beliefs. Cronbach’s alpha of the scale was defined as 0.82. Embarrassment, Dangerousness, Social Dysfunction, and Incurability subscales’ Cronbach’s alpha were 0.69, 0.71, and 0.80, respectively.

The Nurse’s Attitude Towards Forensic Psychiatric Patients Scale was developed to examine the nurses’ attitudes toward forensic psychiatric patients. The validity, reliability, and development study was conducted by Turkish researchers. It has been stated that the scale has validity and reliability (11). Reliability and validity studies of the scale showed the following: content validity index: 0.69; Cronbach’s alpha internal consistency coefficient: 0.86; internal cluster coefficient: 0.86; test-retest reliability coefficient: 0.69. It is a 5-point Likert-type scale (scored between 0 to 5) with 25 items. Subdimensions are social distance (4 items), trust (8 items), willingness to provide care (7 items), and feeling threatened (6 items). Each positive item is scored on a scale of 1 to 5, from “completely disagree” to “completely agree,” depending on whether nurses agree or disagree with each statement. Higher scores indicate positive attitudes toward forensic psychiatry patients.

### 2.3 Statistical analysis

The continuous variables of the research are listed as follows: current age, weekly working hours, working years in the current clinic, and total working years in nursing. Categorical variables were coded dichotomously, such as marital status (married/unmarried) and having a first-degree relative receiving psychiatric treatment (yes/no). The variables were summarized using frequency distributions. The distribution of variables was evaluated using the Kolmogorov-Smirnov test. Statistical methods were chosen depending on the distribution of the data. Pearson’s chi-square test was used for comparisons between categorical variables. Pearson’s or Spearman’s tests were carried out for the correlation analyses. All statistical analyses were performed with SPSS version 26.0 package program, and the significance level was accepted as p<0.05 (two-tailed).

## 3 Results

In the study, 56.5% of the FPN group was female, and 56.9% of the non-FPN group was female. The mean age of the FPNs was 35.46 ± 7.16, and the mean age of the non-FPNs was 36.28 ± 8.78. See **Table 1** for detailed information. The groups were similar in terms of gender, mean age, working time as a nurse, having a family history of psychiatric illness, having a family member with a diagnosis of schizophrenia, and having received psychiatric treatment before. The weekly working hours of the non-FPN group were significantly longer (p=0.019), and they worked for a significantly longer duration in terms of working years in their current units (p=0.001).

**Table 1:** Sociodemographic and occupational characteristics of the participants.

Features	FPN (n=46)	Non-FPN (n=58)	Statistical analysis
Age (years) (M ± SD)	35.46 ± 7.16	36.28 ± 8.78	U:1248.00, Z:-0.56, p ^mw^:0.57
Gender (% Female)	56.5%	56.9%	x^2 =^ 0.00, p^k^=0.97
Marital status (married %)	76.1%	72.4%	x^2 =^ 0.18, p^k^=0.91
Having a first-degree relative receiving psychiatric treatment (yes %)	30.4%	24.1%	x^2 =^ 0.52, p^k^=0.47
Having a schizophrenia patient in a first-degree relative and/or any relative (yes %)	10.9%	5.2%	x^2 =^ 1.17, p^k^=0.28
Psychiatric treatment history (yes %)	13%	24.1%	x^2 =^ 2.03, p^k^=0.15
Having sufficient knowledge about legal aspects of forensic psychiatry (yes%)	43.5%	0%	
Having motivation about working in forensic psychiatry settings- for non- FPNs- yes%	–	12.06%	
Weekly working hours (M ± SD)	46.98 ± 5.53	50.79 ± 9.18	U:985.50, Z:-2.35, p^mw^:0.02
Working years in the current clinic (M ± SD)	1.92 ± 0.85	4.18 ± 3.64	U:841.000, Z:-3.278, p^mw^:0.00
Total working years in nursing (M ± SD)	12.62 ± 816	14 ± 9.52	U:1242.00, Z:-0.602, p^mw^:0.55

^mw^ Mann Whitney U; M, Mean; SD, Standard Deviation; n, number of cases; %, percentage; p, p value; ^k^Chi Square.

The groups were not significantly different in terms of BTMI-total score (p=0.295) and Dangerousness Subscale (p=0.706). The non-FPN group had higher Embarrassment subscale scores(p=0.008). The FPN group had higher scores on the incurability and Social Dysfunction subscale scores than the non-FPN group (p=0.022). According to the NAFPPS, the FPN group had a higher score with more positive attitudes in terms of the Social Distance subscale (p=.009), Trust subscale (p<.0001), Willingness to Provide Care subscale (p<.0001), and Feeling Threatened subscale (p=.004). The results for the total score of the scale (p<.0001) were similar. The nurses’ perceptions of delinquents were also evaluated with the PTCS. Perceptions of delinquents’ social networks were similar between the groups (p=0.334). However, the FPN group had more positive perceptions of the moral/personal aspects of delinquents (p<.0001) (**Table 2**).

**Table 2:** Comparison of the characteristics of the forensic nurses and non-forensic nurses regarding the scales.

		FPN (N=46)	Non-FPN (N=58)
BTMI-embarrassment subscale	MR	44.17	59.10
	Statistics	U=951.00, z=-2.64, p^a^=0.01	
BTMI-dangerousness	Mean	24.41 ± 4.54	24.86 ± 7.47
	Statistics	F(102)=8.88, t= -0.36, p^b^= 0.72	
BTMI-social disfunction and incurability	Mean	30.89 ± 8.46	26.64 ± 9.81
	Statistics	F(102)= 1.541, t=2.33 p^b^=0.022	
BTMI- total	Mean	56.37 ± 11.63	53.47 ± 16.44
	Statistics	F(102)=6.07, t=1.05, p^b^=0.30	
NAFPPS-social distance	Mean	11.50	9.84
	Statistics	F(102)=1.34, t=2.67, p^b^=0.01	
NAFPPS- willingness to provide care	Mean	27.30	19.12
	Statistics	F(102)=4.76, t=8.66, p^b^ <.0001	
NAFPPS- feeling threatened	Mean Rank	62.11	44.88
	Statistics	U=892.00, z=-2.91, p ^a^<.0001	
NAFPPS-trust	Mean	21.96	17.78
	Statistics	F(102)=7.83, t=4.60, p^b^<.0001	
NAFPPS-total score	Mean	77.67	61.62
	Statistics	F(102)=7.17, t=6.88, p ^b^<.0001	
PTCS- moral/personal aspects	Mean	20.85	24.84
	Statistics	F(102)=0.32, t=-4.35, p ^b^=0.007	
PTCS- social network	Mean Rank	49.32	55.03
	Statistics	U=1187.50, Z=-0.97,p ^a^=0.33	

a Mann Whitney U.

b Independent Sample T-test, NAFPPS; Nurse’s Attitude Towards Forensic Psychiatric Patients Scale, BTMI; Beliefs Toward Mental Illness Scale, PTCS; Perception Towards Criminals Scale.

### 3.1 Difference analysis

In this part of the study, the relationship between all attitudes, beliefs, and perceptions toward patients and the sociodemographic and occupational characteristics of nurses was examined.

In the FPN group, the relationship of gender with the scales was evaluated, and only in terms of perception of moral/personal aspects of delinquents, men had significantly higher scores, indicating more negative attitudes- (U=169.000, z=-2.025, p=.043), no significant difference was found between genders in other measurements.

The relation of marital status on the scales was evaluated; single FPNs had significantly higher scores for perceptions of moral/personal aspects of delinquents, indicating more negative perceptions than for married nurses (U=81.000, z=-2,235, p=0,025).

FPNs who thought they did not have enough knowledge about legal aspects of forensic psychiatry had significantly higher scores compared to those who believed they had sufficient knowledge, indicating more negative beliefs, according to the BTMI-social dysfunction and incurability subscale (U=141.00, z=-2.641, p=0.008) and BTMI-total (U=152.500, z=-2.384, p=0.017) scores.

It was found that the person’s living social environment, having a first-degree relative receiving psychiatric treatment, having a diagnosis of schizophrenia in a first-degree relative and/or any relative, and having the motivation to work in a psychiatric service did not make any difference in terms of all the scales.

In the non-FPN group, it was found that the person’s living social environment, gender, marital status, having a diagnosis of schizophrenia in a first-degree relative and/or any relative, and having the motivation to work in a psychiatric service or non-psychiatric service did not make any difference in terms of attitudes, perceptions, and beliefs towards patients. All non-FPN participants stated that they thought that they did not have enough knowledge about the legal aspect of forensic psychiatry. Therefore, this factor was not analyzed. It was found that non-FPNs who wanted to work in the forensic psychiatry setting had higher scores, indicating more positive attitudes, in terms of NAFPPS total score and subscale scores, namely the feeling threatened (U=58.500, z=-2.881,p=0.003), trust (U=84.500, Z=-2,251, p=0.022), willingness to provide care (U=76.000, z=-2,455, p=0.013) subscales and total score (U=51.000, z=-3.045, p=0.001) compared to those who did not want to work in this setting.

The non-FPNs who had first-degree relatives receiving psychiatric treatment showed statistically significant differences in their attitudes; they had lower scores on the NAFPPS feeling threatened subscale score (U=156.500, z=-2.307, p=0.021), indicating more negative attitudes, and had a significantly higher score on the moral/personal aspects of delinquents (U=136.000, z=-2,699, p=0.007), indicating more negative perceptions.

### 3.2 Correlation analysis

In the FPN group, there was a relationship between the number of siblings and BTMI-embarrassment score (rs= .299, p=.043); the weekly working hours and the perception score of the delinquents’ social networks (p=.030, rs=0.321); the weekly working hours and PTCS total score (p=.047, rs=.294); and the number of children and perception score of the delinquents’ social networks (p=.005, rs=-.407). There was no relationship between age, education status, working duration in the forensic service, and total working duration as a nurse (**Table 3**).

**Table 3:** Spearman’s correlation analysis between socio-demographical variables and the NAFPPS, the PTCS, and the BTMI in the forensic nurse group.

	1	2	3	4	5	6	7	8	9	10	11	12	13	14	15	16	17	18	19
1. Age	.																		
2. Number of siblings	.14	.																	
3. Number of Children	.65**	.04	.																
4. Education status (years)	-.05	.29	.01	.															
5. Weekly hours of work	-.05	.00	-.17	.20	.														
6. Working duration at the current unit	.00	-.08	.16	-.04	-.13	.													
7. Total working duration as a nurse	.91**	.06	.60**	-.08	-.16	.11	.												
8. PTCS-Pers/Moral	-.25	-.05	-.41**	.02	.14	-.20	-.28	.											
9. PTCS-Social network	.08	-.05	.06	-.01	.32*	.26	.07	.23	.										
10. PTCS-Total	-.09	-.03	-.22	.02	.29*	.02	-.12	.79**	.74**	.									
11. BTMI-Dangerouness	-.03	.12	.05	.15	.00	.21	-.10	-.07	-.07	-.02	.								
12.BTMI-Embarrasment	.18	.30*	.14	-.11	.03	.09	.08	.19	.13	.24	.16	.							
13. BTMI-Social Dysfunction and Incurability	-.10	.15	-.19	-.20	-.16	.14	-.12	.34*	.05	.25	.41**	.34*	.						
14. BTMI-total	-.12	.19	-.16	-.09	-.14	.14	-.17	.31*	.00	.24	.66**	.38**	.93**	.					
15.NAFPPS-Social Distance	-.08	-.03	.11	.09	-.11	.06	-.03	-.40**	-.02	-.34*	-.35*	-.38**	-.49**	-.56**	.				
16. NAFPPS-Willingness to Provide care	-.27	.12	-.14	-.12	-.03	.11	-.21	-.04	.03	-.03	-.22	-.24	.01	-.09	.14	.			
17. NAFPPS-Trust	-.16	-.20	-.05	.16	-.04	.01	-.09	-.16	-.25	-.28	-.16	-.19	-.30*	-.31*	.31*	.29	.		
18. NAFPPS-Feeling Threatened	.09	-.00	.02	.22	.09	.07	.20	-.35*	-.15	-.34*	-.18	-.37**	-.64**	-.60**	.32*	.32*	.48**	.	
19.NAFPPS-Total	-.12	-.08	-.00	.12	-.02	.13	-.02	-.33*	-.15	-.35*	-.30*	-.43**	-.53**	-.57**	.60**	.61**	.74**	.78**	.

*Correlation is significant at the 0.05 level (2-tailed) ** Correlation is significant at the 0.01 level (2-tailed).

Statistically significant relationships are highlighted in bold.

In the non-FPN group, a positive relationship between age and BTMI-dangerousness (rs=.293, p=0.026) and number of siblings and NAFPPS trust (rs=-.392, p=0.002) was found. There was no relationship between all scale scores and educational status, weekly working hours, total working duration, and working duration in their current clinic. Considering that the person’s living social environment may affect the beliefs about mental patients’ dangerousness, the living environment was checked and the correlation was examined again. Subsequently, the relationship between age and beliefs about dangerousness disappeared (p=.361) (**Table 4**).

**Table 4:** Spearman’s correlation analysis between socio-demographical variables and the NAFPPS, the PTCS, and the BTMI in the non-forensic nurse group.

	1	2	3	4	5	6	7	8	9	10	11	12	13	14	15	16	17	18	19
1. Age	.																		
2. Number of siblings	.51**	.																	
3. Number of Children	.63^**^	.39^**^	.																
4. Education status (years)	-.25	-.09	-.14	.															
5. Weekly hours of work	-.06	.07	-.22	.11	.														
6. Working duration at the current unit	.47**	.38**	.32^*^	-.05	-.04	.													
7. Total working duration as a nurse	.96**	.50**	.60^**^	-.28*	.00	.53**	.												
8. PTCS-Pers/Moral	-.09	-.01	.05	-.00	.19	.00	-.10	.											
9. PTCS-Social network	-.03	-.16	.04	-.05	.16	.11	-.05	.74**	.										
10. PTCS-Total	-.06	-.05	.04	-.04	.20	.05	-.08	.95**	.90**	.									
11.BTMI-Dangerouness	.29*	.18	.23	.07	-.14	.03	.25	.23	.31*	.27*	.								
12.BTMI-Embarrasment	-.05	.01	-.07	.06	-.18	.09	-.15	-.11	-.07	-.12	.10	.							
13. BTMI-Social Dysfunction and Incurability	.16	.16	.17	.13	-.08	.09	.09	.23	.25	.26*	.66**	.22	.						
14.RYHİOtotal	.22	.16	.19	.11	-.13	.11	.15	.22	.29*	.26*	.85**	.32*	.94**	.					
15.NAFPPS-Social Distance	-.17	-.08	-.07	.13	.03	-.02	-.16	-.21	-.22	-.24	-.38**	-.03	-.31*	-.37**	.				
16. NAFPPS-Willingness to Provide care	.06	-.12	-.01	-.19	-.07	-.03	.05	-.40**	-.34**	-.38**	-.44**	-.03	-.30*	-.39**	.48**	.			
17. NAFPPS-Trust	-.23	-.39**	-.14	-.03	-.16	-.15	-.23	-.28*	-.12	-.22	-.33*	-.06	-.22	-.29*	.37**	.67**	.		
18. NAFPPS-Feeling Threatened	-.15	-.22	.03	-.02	-.18	-.22	-.13	-.46**	-.34**	-.44**	-.30*	.01	-.30*	-.32*	.38**	.45**	.54**	.	
19.NAFPPS-Total	-.12	-.26	-.09	-.07	-.12	-.15	-.13	-.45**	-.35**	-.43**	-.44**	-.01	-.35**	-.41**	.66**	.86**	.84**	.72**	.

*Correlation is significant at the 0.05 level (2-tailed) ** Correlation is significant at the 0.01 level (2-tailed).

Statistically significant relationships are highlighted in bold.

### 3.3 Stigmatization related factors

The analysis conducted on the FPN group revealed several significant findings. For the FPN group, gender, marital status, and having sufficient knowledge of the legal aspects of forensic psychiatry made a difference in the scales. In addition, a significant relationship was found between the number of siblings, the number of children, weekly working hours, and the scales. However, education status, working duration in the forensic settings, total working duration as a nurse, person’s living social environment, having a first-degree relative receiving psychiatric treatment, and having a first-degree relative, or any relative, who is a diagnosed schizophrenia patient was not found to be related to the stigmatization among the FPNs.

The analysis of the non-FPN group indicated that having motivation to work in psychiatric settings and having first-degree relatives who receive psychiatric treatment made a difference in stigmatization. Similarly, age and number of siblings also showed significant relationships with stigmatization. Conversely, factors such as the person’s living social environment, gender, marital status, having a diagnosis of schizophrenia in a first-degree or any relative, and the preference to work in a psychiatric service did not demonstrate any significant differences in terms of attitudes, perceptions, and beliefs towards patients among general nurses. Similarly, educational status, weekly working hours, total working duration, and working duration in the current clinic were not found to be related to stigmatization of patients among general nurses.

## 4 Discussion

Overcoming stigma among professionals is challenging; however, it is critical for forensic nursing (18). First, we must recognize its presence, assess its severity, and identify the contributing underlying factors to deal with it. Various factors may affect the medical professionals’ stigmatization levels, such as the type of psychiatric diagnosis, the understanding of the disorders, the level of negative expectations about the outcome of the treatment, the level of medical knowledge regarding psychiatric disorders, and the experience of having family members and friends with mental health problems (32). Understanding the current attitudes, perceptions, and beliefs of FPNs will contribute to developing this professional relationship.

The study showed that, overall, FPNs have more positive beliefs, attitudes, and perceptions than non-FPNs, with some exceptions. Specifically, FPNs had positive beliefs about having any familial relationships and friendships with psychiatric patients, which they found less embarrassing compared to general service nurses. Furthermore, they generally demonstrated more positive attitudes towards people with mental disorders in terms of social distance, trust, willingness to provide care, and feeling threatened. They had more positive perceptions regarding criminality being related to personality traits. The results show that forensic nurses exhibit less stigmatization of patients than their counterparts in general nursing practice, which could be interpreted as a positive situation in the profession. Despite these positive attitudes, beliefs, and perceptions, FPNs also held some negative beliefs, such as the inevitability of social deterioration and the incurability of mental disorders. FPNs were more pessimistic regarding mental disorders leading to deterioration in social relations and that it is incurable compared to non-FPNs. This result is consistent with the finding in the literature that psychiatric nurses generally have less optimistic views on mental disorder outcomes compared to other healthcare providers (39–41). Forensic nurses have more negative beliefs about mental disorders’ outcomes, which may be related to their frequent observation of the negative consequences of psychiatric disorders in forensic units (such as being involved in a crime and being punished because of crime), and generally, they provide care to treatment-resistant patients. While this study did not directly evaluate the effect of hopelessness and stigmatization on patient treatments, previous research has shown a correlation between negative attitudes and a low quality of psychiatric care (42). It can be said that working on the hopelessness of forensic nurses and providing specialized education about mental disorder epidemiology and outcomes will be beneficial for the treatment of patients.

In recent years, there has been a growing awareness of the detrimental effect of stigma on individuals with mental disorders. There is a large body of research showing that mental health stigma is a significant public health problem (43). Consequently, there has been an increase in the number of studies focusing not only on social stigma but also on stigma among healthcare professionals and mental health professionals. However, there are very few studies on the *factors* related to the stigmatization of patients by mental health professionals. There are publications with different results on this subject. In a study examining stigmatization in 92 Jordanian psychiatry nurses, it was stated that lower education level, young age, and not having special training in psychiatric nursing are related to the stigmatization of patients, but the nurses’ gender did not make a difference in stigma (44). In another study conducted in Qatar, it was stated that being male and having recently graduated were associated with stigma among health professionals (34). Cultural and racial differences may be associated with different outcomes (33, 44, 45). In this sense, our study is essential in terms of being conducted in the Turkish health professional population. In our study, we brought this issue one step further and investigated the factors that may be associated with stigmatization in both forensic psychiatry nurses and general medical nurses.

Our findings regarding general medical nurses are as follows: *(I*). *General nurses who reported a willingness to work in a forensic psychiatry setting* perceived the patients as less of a threat. They had higher trust in patients and were more willing to provide care. Nurses generally regard mental health nursing as an unpreferred career option as shown in the literature (46–49). General nurses and nursing students have a lack of interest in working in psychiatry units and forensic units. Our study signifies that nurses with an interest in forensic psychiatry may provide a more positive and accepting attitude toward patients and will help improve the quality of care. Working only with volunteer nurses and other professionals in mental health units is a useful approach to improving the quality of psychiatric care. *(II). Nurses with psychiatric relatives* had more negative attitudes about feeling threatened and held more negative perceptions regarding the moral/personal aspects of delinquents. In our literature review, we could not find any other study investigating the attitudes of nurses with psychiatric relatives. The result obtained in our study can be interpreted as follows. This relationship was shown on the scale of perceptions towards delinquents. Nurses with psychiatric relatives attributed criminality to personality traits instead of familial traits (did not attribute to their own family). It may be an unconscious defense mechanism (denial). Another result, that nurses with psychiatric relatives feel threatened by forensic psychiatry patients, can be explained by the high amount of familial stigma we see in the families of psychiatric patients. Familial stigma is relatively high among mental health patients (50) *(III). Older nurses* had more positive beliefs about mental health patients’ dangerousness than younger ones. All participants in the general nurse group in the study had no experience working in psychiatry service, but still, working in any medical unit can provide expertise in this regard. Thus, one of the explanations that comes to mind first is that the increase in medical experience with aging may be associated with positive approaches. However, no such relationship was found between working time as a nurse in the profession and positive attitudes. Therefore, this result should not be explained by occupational experience. The social environment where the nurses lived consisted of culturally different people and had different social features. The relationship between age and stigma disappeared in the analysis, which was made by controlling the person’s social environment. With this result, instead of saying that age has a direct effect on stigma, it may be said that the living environment is a confounder here. It is possible that living in rural areas may cause people to feel more anxiety in terms of security in general and, therefore, contribute to strengthening the belief that people with mental problems are more dangerous. *(IV)* An *increased number of siblings* was associated with more positive attitudes of trust toward patients among nurses. Research has not been found specifically examining the relationship between number of siblings and stigma toward psychiatry patients among professionals. This situation could be associated with the diversity of people in crowded families and, finally, more accepting attitudes. People who have grown up in large families may show more positive attitudes towards stigmatized members of society.

The findings of factors related to the stigmatization of people with mental health problems by forensic nurses are as follows: *(I) being male* was associated with more negative perceptions. Although previous studies have reported higher stigmatizing attitudes among male nurses (51), there is also research that suggests no significant gender difference in stigma (14). These divergent findings have been attributed to cultural differences. In our study, we did not find any gender differences in the scales that measured various aspects of stigma, except for the Perceptions Towards Criminals Scale. Specifically, no gender difference was observed in beliefs about psychiatric patients or attitudes toward forensic psychiatric patients. Therefore, based on our results, we can partially say that gender differences are not associated with the stigma associated with psychiatric disorders. However, it is noteworthy that male forensic psychiatry nurses, who frequently interact with forensic patients, exhibited more negative perceptions towards delinquents. We think that this should be taken into consideration in the training of forensic psychiatric nurses. *(II) Having insufficient knowledge about the legal aspects of forensic psychiatry* was associated with more negative beliefs about delinquents. Recent research conducted in the United States supports this finding by suggesting that knowledge level may have a positive impact on stigma (13). Another study conducted with mental health stakeholders in Iran highlighted the importance of raising awareness and providing education to reduce stigma (52). Another study suggested various approaches for educational content, emphasizing interactive interventions such as movie screenings and discussions, interactive education, social communication, and group discussions, all tailored to culturally specific characteristics (53). The finding about the relationship between knowledge and stigmatization is important in that it refers to the reduction of stigmatization with specialized training in legal aspects of forensic psychiatry. Previous studies have reported that stigma can be overcome with education (54). *(III) Being single* was linked to negative perceptions about the moral/personal aspects of delinquents, *(IV) a higher number of siblings* was found to be related to higher embarrassment about mental disorders, and *(V) having a higher number of children* was found to be related to positive perceptions about delinquents’ social networks. To the best of our knowledge, there was no previous study on the relationship between number of children and stigmatization. These findings highlight the influence of socio-cultural factors on the stigmatization of forensic psychiatry patients by professionals. *(VI) Longer weekly working hours* were associated with a more negative perception of delinquents’ social networks. This finding is crucial because it shows that long working hours are related to increased stigmatization tendencies. This relationship was not found in general nurses; longer working duration and stigmatization tendencies seem to be related to each other for forensic nurses, according to our results. In our literature review, there were no studies investigating the relationship between long working hours and stigmatization. However, long working hours may be a predictor of job stressors and burnout (55, 56). Burnout has recently been noted to increase stigma among physicians (57). In this sense, we speculate that burnout may be a mediator for this relationship. This should be accepted as not just a researcher’s opinion but also a rational one. We did not use any questionnaire for burnout in the study, so there is a need for extended research on burnout and stigmatization. We discussed this in the context of the relationship between longer working hours and increased stress and burnout. Although this relationship was not found in general nurses, burnout and stigma seem to be parallel to each other in forensic nurses according to our results. In light of this information, burnout in forensic nurses should be considered. While academic evidence partially supports the findings of our study, we could not verify the findings due to limited research in this discipline. More research focusing on nurses and their stigmatization of people with a mental health condition is needed to provide more comprehensive and specific evidence.

Contrary to previous studies, it may be confusing that many factors were found to be associated with stigma in both the forensic nurse group and the general nurse group in this study. Unlike previous studies that primarily assessed attitudes using a single scale, we evaluated different essential aspects of stigma, including beliefs, attitudes, and perceptions, at the same time. Using various scales for the measurement of stigmatization explains the high number of factors that we found to be associated. This methodology is particularly valuable as it offers a more comprehensive understanding of the complex nature of stigma in this context.

Stigma and culture are intertwined and must be evaluated at the same time; thus, when planning research about stigma, it is crucial to choose appropriate methods for the population. Therefore, this study used standardized measurement tools for the Turkish population. Our study provides clear and new information about the stigmatization by health professionals, especially forensic psychiatry nurses, of mental patients. While these results enable the identification of problematic areas in nursing care, they also guide the development of culture-specific interventions to reduce stigma.

### 4.1 Limitations and suggestions for future studies

The small sample size is an essential limitation of the study. The small sample size may lead to lower study power and false negative outcomes. We may not have been able to demonstrate all existing relationships in this context. Therefore, there is an evident need for research with larger sample sizes. By the time we constituted the sample of forensic psychiatric nurses, we had tried to restrict the use of established high-security forensic units in our country since 2018. We excluded nurses from regional mental health hospitals’ forensic services because these hospitals do not distinguish between forensic and non-forensic nurses. This exclusion was made considering that nurses have more experience with forensic psychiatric patients than general psychiatric nurses. As a consequence, our study encountered a limitation in sample size. However, we find the results valuable due to our country’s rarity of forensic psychiatric nurses. This will stimulate further research on stigmatization among this specific group of healthcare professionals in Turkiye.

Additionally, the forensic nurse group in our study comprises forensic nurses from the same institution, which is a consequence of the limited number of available FPNs. Consequently, the findings of our study may only be generalized to some of the population. Moving forward, future research examining stigma in heterogeneous groups will be invaluable. Moreover, while we emphasized the importance of education, we did not directly evaluate its effectiveness, which means we could not exclude the influence of confounding factors. In future studies, conducting group comparisons on stigmatization after specific training will be valuable in demonstrating the effectiveness of education.

## 5 Conclusions

Stigmatization among forensic psychiatric nurses is a lesser-explored area in research; however, its significance should not be understated. It would be beneficial to conduct multi-center studies with large samples and focus on the effects of education level and specific training in the future. The study showed that forensic psychiatric nurses exhibit lower levels of stigmatization of patients compared to general practice nurses. Furthermore, it identified several factors related to stigmatization among nurses. For forensic psychiatric nurses, these factors included gender, marital status, sufficient knowledge of the legal aspects of forensic psychiatry, number of siblings, number of children, and weekly working hours. Similarly, among general practice nurses, factors such as motivation to work in psychiatric settings, having first-degree relatives receiving psychiatric treatment, age, and number of siblings were found to be associated with stigmatization of patients

The findings highlight the importance of understanding and addressing stigmatization by health professionals, particularly in forensic psychiatric nursing. Identifying the current attitudes, perceptions, and beliefs of forensic nurses will provide more advanced treatment in forensic psychiatric care and supportive and empathetic professional relationships between patients and professionals. Additionally, the study provides valuable insights into stigmatization among general nurses, emphasizing the need for interventions in general health care that serve people with a mental health condition. Educating all nurses, especially those working with forensic psychiatric groups, about mental illnesses during pre-vocational and in-service training processes, and as a result, preventing possible stigmatizing attitudes, will increase the quality of healthcare.

## Data Availability

The raw data supporting the conclusions of this article will be made available by the authors, without undue reservation.
